# A severe influenza season in Austria and its impact on the paediatric population: mortality and hospital admission rates, november 2017 - march 2018

**DOI:** 10.1186/s12889-020-8239-2

**Published:** 2020-02-04

**Authors:** Benno Kohlmaier, Vendula Svendova, Thomas Walcher, Heidemarie Pilch, Larissa Krenn, Herbert Kurz, Karl Zwiauer, Werner Zenz, for the Austrian Paediatric Influenza Network, Günther Bernert, Robert Birnbacher, Walter Bonfig, Robert Bruckner, Doris Ehringer-Schetitska, Josef Emhofer, Reinhold Kerbl, Gerhard Pöppl, Josef Riedler, Hans-Robert Salzer, Klaus Schmitt, Andreas Wimmer

**Affiliations:** 1 Department of General Paediatrics, Medical University of Graz, Graz, Austria; 2 Institute for Medical Informatics, Statistics and Documentation, Medical University of Graz, Graz, Austria; 3 Department of Paediatrics and Adolescent Medicine, Medical University of Graz, Graz, Austria; 4 Department of Paediatrics and Adolescent Medicine, Social Medical Center East - Danube Hospital (SMZ-Ost), Vienna, Austria; 5 Department of Paediatrics and Adolescent Medicine, University Hospital St. Pölten, St. Pölten, Austria

**Keywords:** Influenza, Children, Hospital admission rates, Mortality, Vaccination, Austria

## Abstract

**Background:**

In Austria paediatric influenza-associated hospitalisations and deaths have never been systematically monitored. We examined the influenza season 2017/18 in terms of hospitalisation and mortality in the Austrian paediatric population and put the results into perspective of the available data from the last 15 years.

**Methods:**

Cases of influenza-associated hospitalisations and deaths for season 2017/18 in children below 18 years were retrospectively collected from 12 and 33 Austrian hospitals, respectively. Hospitalisation and mortality rates for the whole Austrian paediatric population were estimated, adjusting for the population in each catchment area. Two Austrian databases were queried for hospitalisations and deaths associated with influenza during 2002–2016. Rough estimate of the vaccination coverage was calculated from a survey on 100 inpatients.

**Results:**

Influenza-related paediatric hospitalisation rate in season 2017/18 was estimated as 128 (CI: 122–135) per 100,000 children, much higher than the national average of 40 per 100,000 over the years 2002–2016. There were nine reported influenza-associated deaths among children, resulting in mortality rate of 0.67 (CI: 0.32–1.21) per 100,000 children.

**Conclusions:**

Reported influenza-associated hospitalisations and fatalities demonstrate a high burden of influenza in the Austrian paediatric population corresponding with very low vaccination coverage.

## What is known:

- Children are among the most affected by the seasonal influenza outbreaks worldwide.
- Vaccination coverage against influenza in Austrian children is very poor (last reported below 5%).
- There is no active surveillance of influenza associated hospitalisations and mortality in Austria.

## What is new:

- This work is the first active monitoring of paediatric influenza associated hospitalisations and mortality in Austria.
- An analysis of data from two Austrian databases showed a median number of paediatric influenza-associated deaths of 1 (ranging from 0 to 4 cases) over the years 2002–2016.
- An especially severe influenza season 2017/18 resulted in more than 2000 hospitalisations and nine paediatric deaths.

## Background

Seasonal influenza is an infectious disease caused by influenza viruses, causing a high rate of deaths and hospital admissions worldwide [1]. Children are among the most affected during the annual outbreaks: a recent meta-analysis estimated that one in ten of all unvaccinated children is infected by seasonal influenza every year [2].

Hospitalisation and mortality in paediatric patients with influenza represent only a small proportion all influenza cases [3] Both are widely influenced by patient specific factors like age and comorbidities [4, 5].

Large amount of literature on hospitalisation and mortality rates focuses on the United States [6–12]. Latest study reported annual mortality of 0.15 per 100,000 children [13]. In Europe, several country-based studies reported influenza-related paediatric hospitalisation or mortality rates [14–17], but these data are lacking in many other European countries, including Austria.

In Austria, mortality rates due to influenza during 2001–2009 were estimated to be 15.5 deaths per 100,000 people of all ages [18]. Despite several Austrian agencies monitoring cases of influenza-like illness, such as Austrian Agency for Health and Food Safety (AGES) or Diagnostic Influenza Network Austria (DINÖ), there are no published data on paediatric hospitalisation and mortality due to influenza.

Since 2013 influenza vaccination is recommended for all children from 6 month of age in Austria [19]. However, general vaccination recommendation is not linked to governmental funding for vaccination and influenza vaccination is not covered by the general health insurance. Information about influenza vaccination rates are derived from numbers of sold vaccines and implicate a very low vaccination rate [20].

In our study, we aimed to systematically monitor influenza-associated pediatric deaths and estimate influenza-associated pediatric hospitalisations to raise the awareness of the burden of paediatric influenza. We estimated influenza-related hospitalisation rates based on retrospectively collected data from Austrian paediatric hospitals which participated in our survey and compared the findings to the data from the years 2002–2016. We described prospectively collected inpatients from the Department of Paediatric and Adolescent Medicine, Medical University of Graz, in greater detail, including crucial clinical parameters. We reported on influenza-associated fatalities among children during 2017/18, collected prospectively, and compared to the historical data from 2002 to 2016, collected retrospectively. Our secondary aim was to obtain a rough estimate of vaccination coverage, by conducting a survey among the general inpatients in the paediatric hospital in Graz.

## Methods

To assess hospitalisation and mortality rates in Austria, we prospectively recorded the data from our local hospital in Graz, and sent a survey asking for influenza related hospitalisations and deaths to all 50 paediatric hospitals listed by the Austrian Society of Paediatrics and Adolescent Medicine. Data from the hospitals that replied to our survey were then extrapolated to the whole Austrian population. Patient’s age range was set to 0–17 years according to the Austrian law, which states that patients younger than 18 years should be admitted to paediatric hospitals, whereas older patients should be admitted to adult hospitals.

### Clinical description of inpatients with influenza at the Department of Paediatrics and Adolescent Medicine, Medical University of Graz, Austria, 2017/18

All paediatric inpatients at the Department of Paediatric and Adolescent Medicine, Medical University of Graz aged less than 18 years with virologically confirmed influenza infection were prospectively recorded during the influenza season from the 1st of November 2017 till the 31st of March 2018. All patients were reviewed by a physician and classified according to ECDC (European Centre for Disease Prevention and Control) case definition [21] and their clinical data were documented. Influenza infection was confirmed either by rapid antigen test with nasopharyngeal swabs (sensitivity 66.1% and specificity 98.3% for children [22]**,** or by PCR from blood. The choice of test was based on clinician’s judgement. Since the vaccination status of inpatients had been recorded incompletely during their hospital stay, a telephone survey was conducted after discharge asking the parents about the influenza vaccination history of their children.

### Estimation of influenza-associated hospitalisations in Austria, 2017/18

We aimed to obtain hospitalisation rates from all 50 Austrian paediatric hospitals, nevertheless, we did not get response from all of them and had to estimate the nation-wide hospitalization rates. We used the information system of the Austrian Health Research Institute, Gesundheit Österreich Forschungs- und Planungs GmbH (GOEG), to find out the number of paediatric inhabitants in the catchment area of each hospital. We estimated the total number of paediatric inpatients with influenza infection in 2017/18 using Poisson regression with the population of each catchment area as an offset variable, and reported the 95% confidence interval (CI). We had to resort to using the population data from 2017 as seasonal population data were not available. We calculated the hospitalisation rate per 100,000 children as 100,000 ∗ (*h*/*N*), where *h* was the estimated number of hospitalized influenza patients and *N* the Austrian population under 18 years of age.

### Influenza-associated paediatric mortality in Austria, 2017/18

To investigate the total number of paediatric influenza-associated deaths in Austria for the season 2017/18, an email survey was conducted among all 50 paediatric hospitals in Austria. The survey included age, sex, relevant chronic conditions, influenza subtype, vaccination status and cause of death. In centres with lethal cases, a treating physician was asked to review the cases. As we did not get a response from all the hospitals, we again estimated the total number of lethal cases using Poisson regression with the population of each catchment area as an offset variable, and reported the 95% confidence interval (CI). We calculated the mortality rates per 100,000 children as 100,000 ∗ (*d*/*N*), where *d* was the estimated number of lethal cases and *N* the Austrian population below 18 years of age (in 2017).

### Influenza-associated paediatric hospitalisation and mortality rates in Austria, 2002–2016

For comparison with previous seasons, we used data from GOEG once again to find the number of paediatric influenza-associated inpatients and number of deaths for each year from 2002 to 2016. GOEG queried two databases - Statistics Austria and the Federal Ministry of Labour, Social Affairs, Health and Consumer Protection (BMASGK) - using the following search criteria: cause of death with ICD-10 coding J09, J10 and J11 as main or secondary diagnosis. In case of discrepancy in death counts between the two databases, the higher number was taken.

### Estimation of paediatric vaccination coverage in Austria 2017/18

The latest published estimates of paediatric vaccination coverage in Austria, 3.43 and 4.30%, were described for the season 2010/11 [17, 18]. Austria did not provide the data to ECDC for the latest seasons, hence we have no information about the current vaccination coverage. In order to obtain at least a rough estimate of more recent vaccination rates, clinicians in our hospital randomly asked parents of 100 paediatric inpatients older than 12 months and younger than 18 years (hospitalized for any reason except for influenza), whether their children were vaccinated against influenza, and, for comparison, tick-borne encephalitis (TBE) and measles, mumps and rubella (MMR). The confidence interval of a child being vaccinated was constructed using exact binomial test.

## Results

### Clinical description of paediatric inpatients with influenza at the Department of Paediatrics and Adolescent Medicine, Medical University of Graz, Austria, 2017/18

**Fig. 1:**
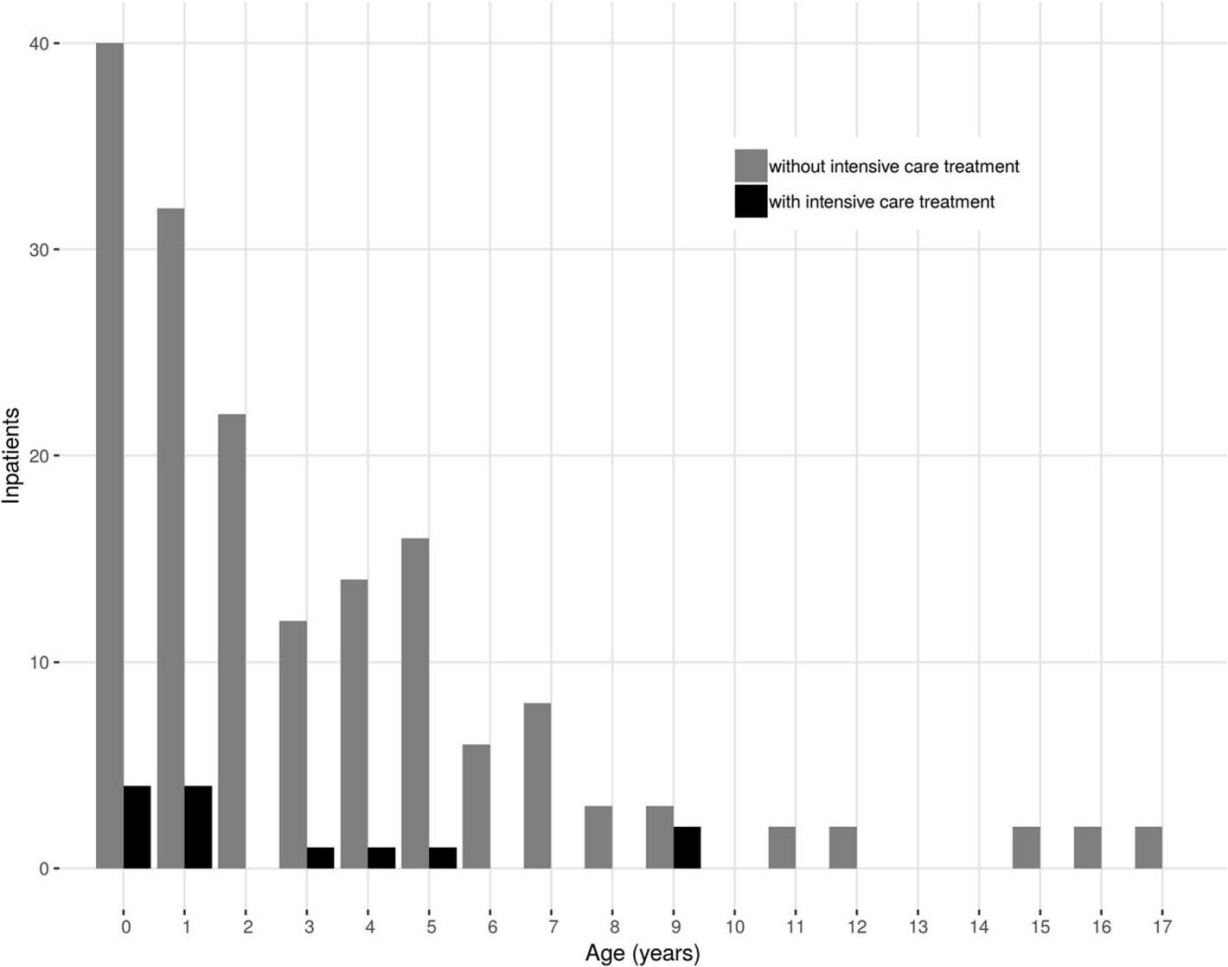
Age distribution of influenza-related paediatric hospitalisations in Graz, Austria 2017/18

From November 1st 2017 to March 31st 2018 a total of 708 paediatric patients with virologically confirmed influenza infection were seen at the Department of Paediatric and Adolescent Medicine, Medical University of Graz. These patients had a total of 800 outpatient visits (1.13 visits per patient). 166 (23% of all patients) were admitted to the ward (for inpatient characteristics and age distribution see Table 1 and Fig. 1, respectively). One hundred forty-five patients (89%) were positively diagnosed via rapid antigen test, 17 (10%) via PCR and 4 patients (1%) via both of these tests. We were able to reach parents of 125 inpatients (75%) for a query about vaccination against influenza. Only one of these 125 patients had been vaccinated against influenza in the season 2017/18. Reasons for hospital admission included high fever (32%), dyspnea (19%), febrile seizure (12%), dehydration (10%) and diarrhea or vomiting (9%). Rare reasons included sepsis, suspected meningitis, myocarditis, myositis, hematemesis, reaction to oseltamivir, and reduced general condition. 23 (15%) inpatients had an underlying chronic condition including 8 patients with developmental delay. Nine children (5.4% of all inpatients) needed intensive care treatment including one child who died from acute necrotizing encephalitis and three patients who had chronic conditions, in particular chronic kidney disease, De-Morsier-Syndrome and spastic quadriplegia. Two patients needed invasive ventilation for a total of nine and ten days, and two patients needed non-invasive ventilation (Optiflow™ | Fisher & Paykel Healthcare).

**Table 1:** Characteristics of 166 inpatients with virologically confirmed influenza infection of the Department of Paediatric and Adolescent Medicine, Medical University of Graz, Austria, from November 2017 to March 2018

Number of patients	166
Age: median (IQR)	2 years (1–5)
Gender (male)	94 (57%)
Subtype - A	112 (68%)
Subtype - B	49 (30%)
Subtype - A + B	2 (1%)
not known	3 (2%)
Hospital stay: median (IQR)	2 days (2–4)
Paediatric intensive care unit (PICU)	9 (5%)
Hospital stay on PICU: median (IQR)	6 days (3–8)
Antibiotics on PICU	8 (89% of PICU patients)
Invasive ventilation	2 (1%)
Non-invasive ventilation	2 (1%)
Oxygen therapy	10 (6%)
Max. CRP (mg/l): median (IQR)	8.2 (2.95–21.20)
Leukocytes (G/l): median (IQR)	7.3 (5.4–10.6)
Antibiotics before admission	16 (10%)
Antibiotics on ward	38 (24%)
Deaths	1 (1%)
Vaccinated	1 out of 166

*CRP* C-reactive protein, *IQR* interquartile range

### Estimation of influenza-associated paediatric hospitalisations in Austria, 2017/18

We contacted 50 Austrian paediatric hospitals in order to obtain the numbers of influenza inpatients for the 2017/18 season. However only 12 (24%) hospitals responded to our survey and reported their counts (see Table 2). Due to the geographical distribution of the hospitals, located in north, middle and south parts of Austria, we consider these hospitals to be a representative subsample of all paediatric hospitals in Austria. We estimated the absolute number of influenza inpatients in Austria for the 2017/18 season to be 2072 (CI: 1968–2179). Hospitalisation rate was then 128 (CI: 122–135) per 100,000 children.

**Table 2:** Reported numbers of influenza-associated inpatients from 12 Austrian paediatric hospitals and population below 18 years of age in their catchment area from November 2017 to March 2018

Hospital	Influenza inpatients	Population
Graz LKH	166	147,715
Wien SMZ OST Donauspital	129	78,046
Linz BSRV KH	81	52,895
St. Pölten UnivKL	64	41,010
Leoben-Bruck/Mur LKH	62	50,557
Steyr LKH	56	21,221
Wien SMZ SÜD KFJ/Preyer	46	45,724
Wels-Grieskirchen KL	38	47,206
Ried/Innkr BSRV KH	32	23,103
Tulln UnivKL	31	31,708
Kirchdorf/Krems LKH	25	10,942
Oberwart LKH	12	29,707
*Total*	*742*	*579,834*

### Influenza-associated paediatric deaths in Austria, 2017/18

All 50 paediatric hospitals were contacted and 33 replied. There were nine reported lethal cases, five males and four females, median age of 4 years. Three children had no underlying disease, one had a congenital autoinflammatory syndrome and five neurodevelopmental delay. Cause of death was in four cases acute respiratory distress syndrome, in two cases elevated intracranial pressure, in two cases asphyxia and in one case pneumonia. Subtype A was the most prevalent type of influenza virus (7 patients–78%), one patient had subtype B and one had both subtypes positive. See Table 3 for all relevant characteristics of the cases. Vaccination status was available from six children and none of them was vaccinated against influenza. We estimated the absolute number of influenza-related deaths inpatients in Austria for the 2017/18 season to be 11 (CI: 5–20). Mortality rate was then estimated as at least 0.67 (CI: 0.32–1.21) per 100,000 children.

**Table 3:** Characteristic of influenza-associated paediatric deaths in Austria in the influenza season from November 2017 to March 2018

Age (years)	Sex	Subtype	Relevant chronic conditions	Vaccinations against influenza	Cause of death
3	m	A + B	trisomy 21	not vaccinated	cerebral abscess
3	f	A	none	not vaccinated	acute stenosing laryngotracheitis
3	m	A	developmental delay	not vaccinated	ARDS
4	m	A	autoinflammatory syndrome	not vaccinated	pneumonia
4	f	A	tetraspastic paresis	not known	ARDS
4	f	A	none	not vaccinated	cerebral pressure
5	m	A	Bardet-Biedl syndrome	not known	ARDS
10	f	A	tetraspastic paresis	not vaccinated	ARDS sepsis
12	m	B	asthma	not known	severe asthma

*ARDS* acute respiratory distress syndrome, *f* female, *m* male

### Influenza-associated paediatric hospitalisation and mortality rates in Austria, 2002–2018

**Fig. 2:**
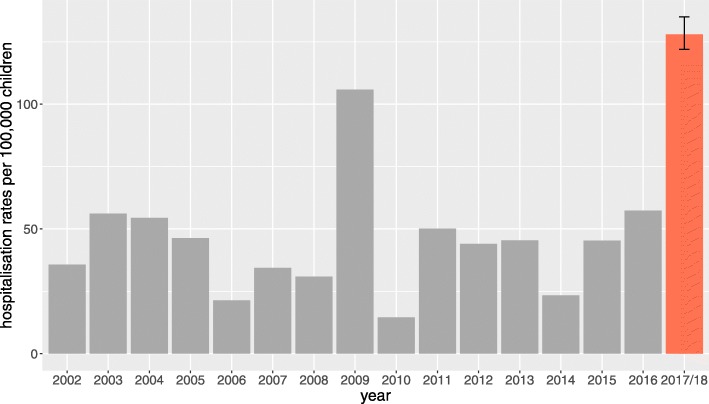
Influenza-associated paediatric hospitalisation rates in Austria 2002–2018. Data 2002–2016 collected passively by searching ICD-10 codes, data 2017/18 estimated from counts actively collected via a survey

**Fig. 3:**
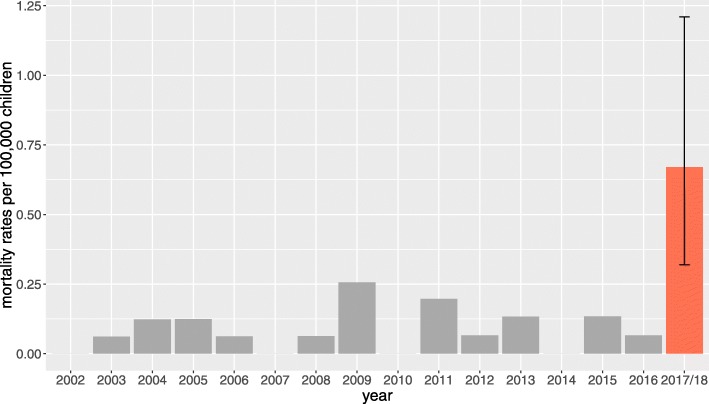
Influenza-associated paediatric mortality rates in Austria 2002–2018. Data 2002–2016 collected passively by searching ICD-10 codes, data 2017/18 estimated from counts actively collected via a survey

Database search results from GOEG showed a median of 675 (ranging from 224 to 1653 cases) influenza paediatric inpatients and median of 1 (ranging from 0 to 4 cases) influenza-associated deaths over the years 2002–2016. Hospitalisation and mortality rates were calculated per 100,000 children (Table 4) and depicted on Figs. 2 and 3. The comparison of both databases we queried for number of deaths (Statistics Austria and BMASGK) are shown in Table 5.

**Table 4:** Influenza-associated paediatric inpatients and deaths in Austria between 2002 and 2018. The number of inpatients and deaths in Austria during season 2017/2018 was estimated from reported cases in 12 and 33 Austrian hospitals, respectively, while the cases in 2002–2016 were collected retrospectively by searching ICD-10 codes

Year	Number of Inpatients	Number of inpatients per 100,000 children	Number of deaths	Number of deaths per 100,000 children
2002	582	35.7	0	0.00
2003	912	56.2	1	0.06
2004	880	54.4	2	0.12
2005	747	46.3	2	0.12
2006	344	21.4	1	0.06
2007	548	34.4	0	0.00
2008	487	30.9	1	0.06
2009	1653	105.8	4	0.26
2010	224	14.5	0	0.00
2011	764	50.1	3	0.20
2012	665	44.1	1	0.70
2013	681	45.4	2	0.13
2014	349	23.4	0	0.00
2015	676	45.3	2	0.13
2016	867	57.3	1	0.70
11/2017 03/2018	2072 (CI: 1968-2179)	128 (CI: 122–135)	11 (CI: 5–20)	0.67 (CI: 0.32–1.21)

**Table 5:** Influenza-associated paediatric deaths in Austria between 2002 and 2016 – comparison of two databases

Year	Number of deaths according to BMASKG^a^	Number of deaths according to Statistics Austria
2002	0	0
2003	1	1
2004	2	0
2005	2	0
2006	1	0
2007	0	0
2008	1	0
2009	4	3
2010	0	0
2011	3	3
2012	1	0
2013	1	2
2014	0	0
2015	1	2
2016	1	1

^a^ Federal Ministry of Labour. Social Affairs, Health and Consumer Protection

### Estimation of vaccination coverage in Austria 2017/18

The survey among inpatients in the paediatric hospital in Graz showed that only three out of 100 patients were correctly vaccinated against influenza, while 85 against TBE and 90 against MMR. The probability of a child being vaccinated against influenza was estimated as 0.03 with 95% confidence interval (0.016–0.113).

## Discussion

In this study, we describe the influenza season 2017/18 in Austria, resulting in nine reported paediatric influenza-associated deaths and approximately 2072 hospitalisations of children. We showed that the calculated paediatric hospitalisation and mortality rates (128 and 0.67 per 100,000 children, respectively) were several times above the national average, calculated over the last 15 years.

It is important to acknowledge the differences in data collection for years 2002–2016 (retrospective) and season 2017/18 (prospective), hence possible underestimation of the counts in the 2002–2016 time range. The conclusions of our results are, nevertheless, supported by the reports of exceptionally severe influenza season 2017/18 in Austria [23] as well as in Germany [24]**,** the whole of Europe [25] and the United States [26].

The observed number of paediatric influenza-associated deaths during the season 2017/18 exceeded the number of the Austrian paediatric deaths in the year 2017 caused by the following vaccine preventable diseases combined: measles, rubella, hepatitis A, hepatitis B, poliomyelitis, pertussis, diphtheria, and infections with *Streptococcus pneumoniae*, *Neisseria meningitidis* and *Haemophilus influenzae* [unpublished data from GOEG].

A recent study estimated that vaccination against influenza could reduce the paediatric mortality by 50% or by 65% in children with or without an underlying disease, respectively [11]. Despite Austria’s recommendations to vaccinate all people older than 6 months against influenza, the country has been described as resistant against influenza prevention and control [27]. Also, there is little information about influenza vaccination rates in the Austrian population. The European Centre for Disease Prevention and Control (ECDC) published a report on influenza vaccination rates during seasons 2007–2008, 2014–2015 [28] and 2015–16, 2016–17 [25]. Austria was among the few European countries recommending influenza vaccination for all children older than 6 months [19], yet in both ECDC reports failed to provide an estimation of vaccination coverage for the study. The latest published estimates of paediatric vaccination coverage in Austria were approximately 4% for season 2010/11 (3.43 and 4.3% [29, 30]), the lowest coverage among all age groups. There is no other more recent published source of vaccination rates in Austrian children.

Our survey on the influenza vaccination status at the Department of Paediatric and Adolescent Medicine Graz showed only 3 out of 100 children as being vaccinated according to vaccination recommendations. This suggests that the vaccination coverage has not increased since the latest report from 2011 [30].

In comparison, the same survey showed that 85 out of 100 children were correctly vaccinated against tick-borne encephalitis and 90 against measles, mumps, and rubella (MMR). This implies that the Austrian public is highly susceptible to governmental vaccination recommendations for preventable diseases.

The failure to increase the vaccination coverage for influenza in Austrian adults has been ascribed to ignorance of the health care system, a lack of social marketing and, as a consequence, an underestimation of the seriousness of the disease by the general public [31].

### Limitations

Our study has several limitations. Only 24 and 66% of paediatric hospitals responded to our survey on hospitalisation and mortality, respectively, hence we had to estimate the total number of inpatients and deaths, reporting the 95% confidence interval as a variability measure.

Another limitation is that the frequency of testing for influenza viruses might differ between hospitals, resulting in a reduced detection rate of influenza cases. Also, when comparing season 2017/18 to the previous years, it is important to point out that the data collection for the years 2002–2016 was performed passively by searching for ICD-10 codes, whereas the data for season 2017/18 were collected actively via a survey. Therefore, a direct comparison of differences between 2002 and 2016 and season 2017/18 might be exaggerated. It remains unclear if the lower number of inpatients and lower death rate in previous seasons were due to underreporting or due to a milder course of disease. Despite this limitation, we show the comparison for argument’s sake.

The conducted survey about the vaccination rate of inpatients is limited by the small number of participants, the preselected participants including only patients treated at our hospital and the exclusion of infants. Despite the limitations of the survey, we included its results to have a rough estimate of vaccination rates, since there is no publication available about actual influenza vaccination rates in children in Austria.

Here we describe patients with influenza not regarding other co-infections including other respiratory diseases. Co-infections are common and in some cases the severity of diseases and need for inpatient treatment could be influenced by the combination of pathogens.

## Conclusion

In summary, our data from the severe influenza season of 2017/18 emphasize the burden of paediatric influenza in Austria. We suggest a paediatric influenza surveillance network to be established, providing continuous reports and easy access to the public, similar to the one in the United States [32]. Such surveillance should include diagnosed paediatric cases, paediatric hospital admissions, patients requiring intensive care, and paediatric influenza-associated deaths.

## Data Availability

The datasets generated and/or analysed during the current study are not publicly available due to individual patient protection but are available from the corresponding author on reasonable request.

## Abbreviations

AGES: Austrian Agency for Health and Food Safety
BMASGK: Federal Ministry of Labour, Social Affairs, Health and Consumer Protection
DINÖ: Diagnostic Influenza Network Austria
ECDC: European Centre for Disease Prevention and Control
GOEG: Gesundheit Österreich Forschungs- und Planungs GmbH
ICD: International Statistical Classification of Diseases and Related Health Problems
IQR: Interquartile range
MMR: Measles, mumps and rubella
PCR: Polymerase chain reaction
TBE: Tick-borne encephalitis
